# Supplementary material for: Effects of ageing and frailty on circulating monocyte and dendritic cell subsets
Source: NPJ Aging. 2024 Mar 4;10(1):17. doi: 10.1038/s41514-024-00144-6 (PMC10912203; doi:10.1038/s41514-024-00144-6)
Supplement: Supplementary file 1 — Supplemental material [file 41514_2024_144_MOESM1_ESM.pdf]

Supplementary data

Supplementary table 1: the 14 antibodies used in the flow cytometry panel

**Marker**	**Fluorochrome**	**Company**	**Catalogue number**
CCR7	PE	BD Biosciences	552176
CD1c	BUV395	BD Biosciences	742751
CD11c	APC	BD Biosciences	333144
CD14	Pacific Orange	Life Technologies	MHCD1430
CD16	BUV737	BD Biosciences	564434
CD19	AF-700	Thermo Fisher Scientific	56-0199-42
CD40	APC-Cy	BioLegend	334224
CD86	BB515	BD Biosciences	564544
CD141	BB700	BD Biosciences	742245
CD303	BV785	BioLegend	354222
HLA-DR	V450	BD Biosciences	655874
PD-L1	PE-Cy7	BioLegend	329715
TLR2	BV650	BD Biosciences	742769
TLR4	BV711	BD Biosciences	564404

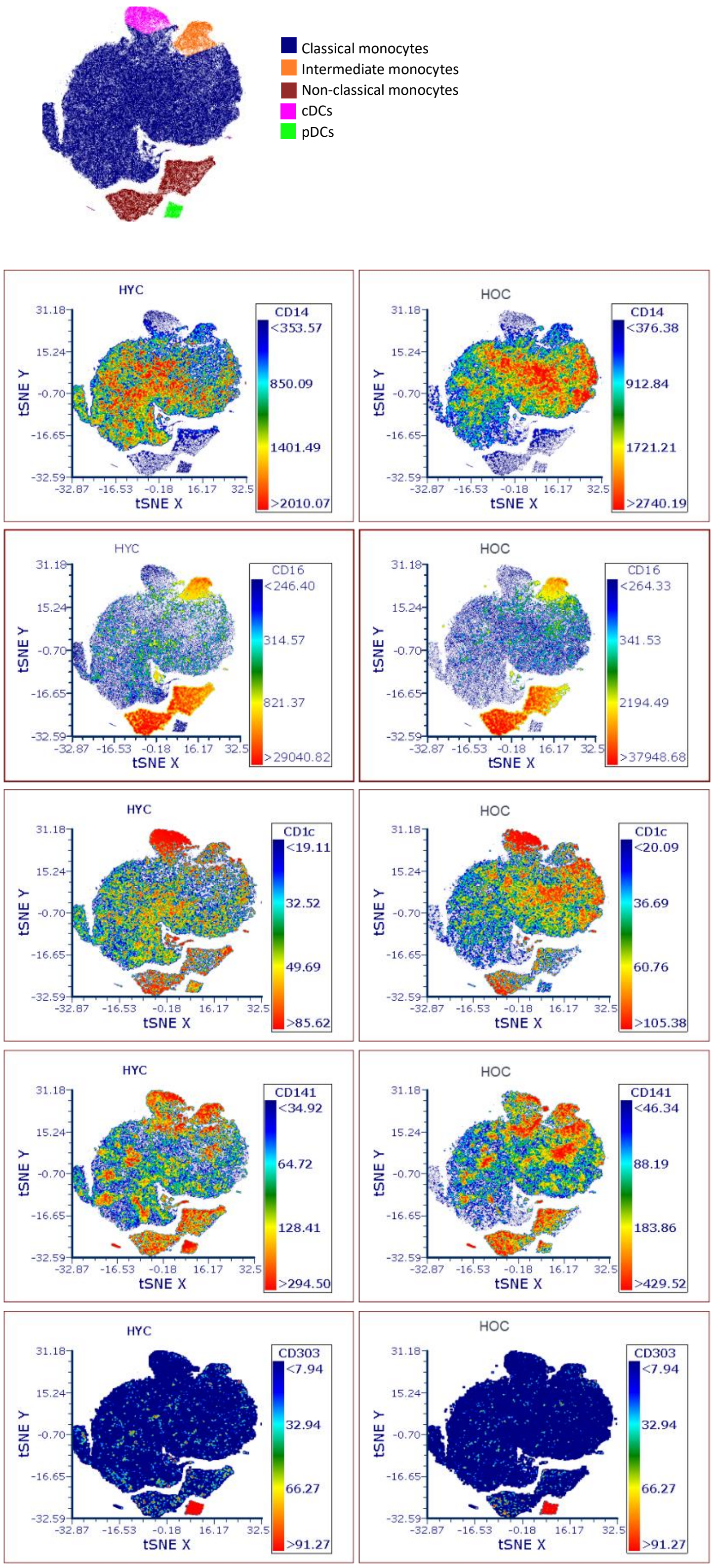

Supplementary figure 1: Expression of lineage markers CD14, CD16, CD1c, CD141 and CD303 in t-SNE plots of healthy young controls (HYC) and older healthy controls (HOC) for the identification of clusters with non-classical monocytes (CD14lowCD16+), intermediate monocytes (CD16+CD14+), classical monocytes (CD14+CD16-), conventional dendritic cells (CD141/CD1c+) and plasmacytoid dendritic cells (CD303+). t-SNE: t-distributed Stochastic Neighbor Embedding.

A

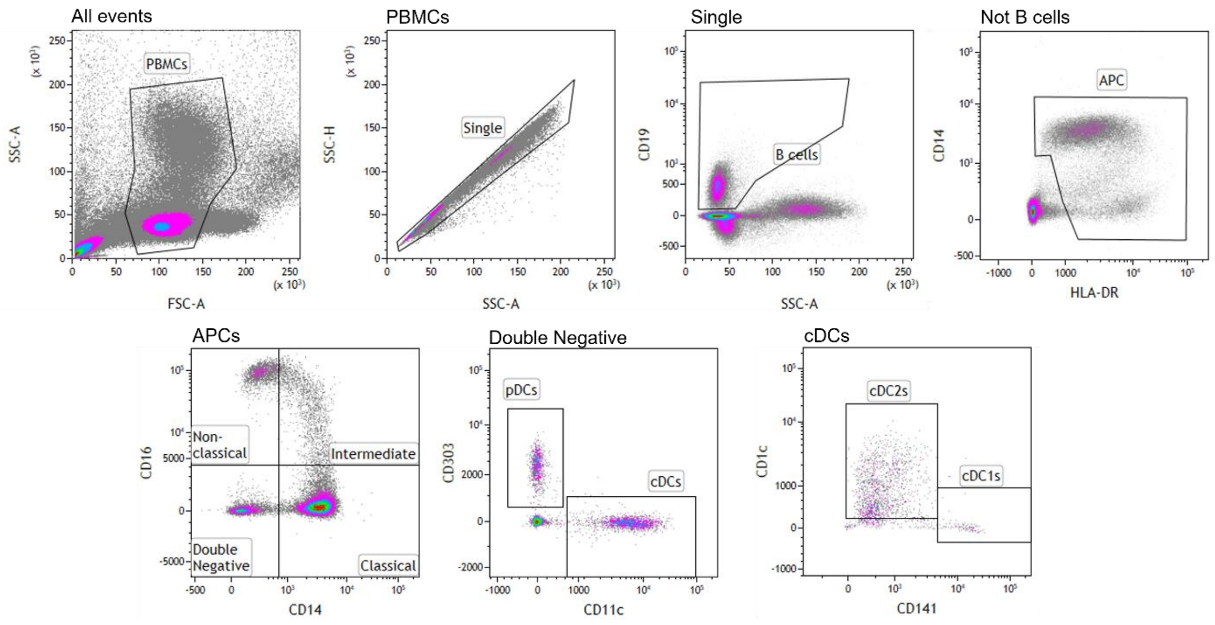

B

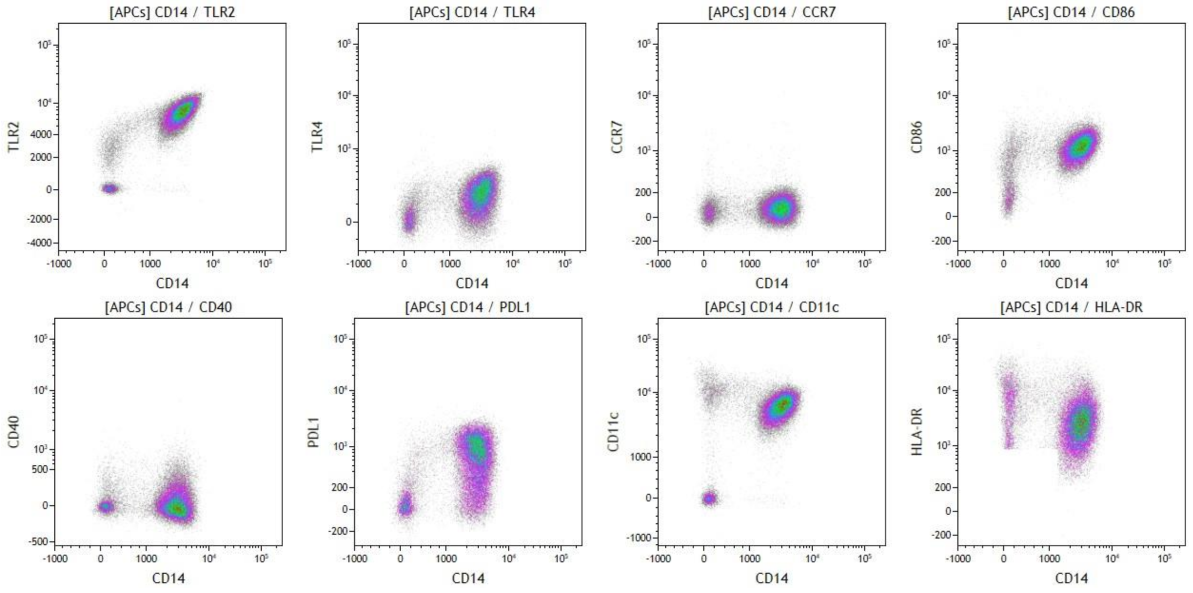

Supplementary figure 2: Gating strategy and marker expression in the flow cytometry experiments. A: Single PBMCs were selected based on size and granularity. CD19 was used to gate out B cells. CD14 and HLA-DR were used to select all antigen presenting cells. Antigen presenting cells were further classified into non-classical, intermediate and classical monocytes based on CD16 and CD14 expression. CD16/CD14 double negative cells were divided into pDC based on CD303 and cDCs based on CD11c expression. cDC2 cells were CD1c positive and cDC1 cells were CD141 positive. B: Example staining of the expression of TLR2, TLR4, CCR7, CD86, CD40, PDL1, CD11c, HLA-DR against CD14 in total APCs.

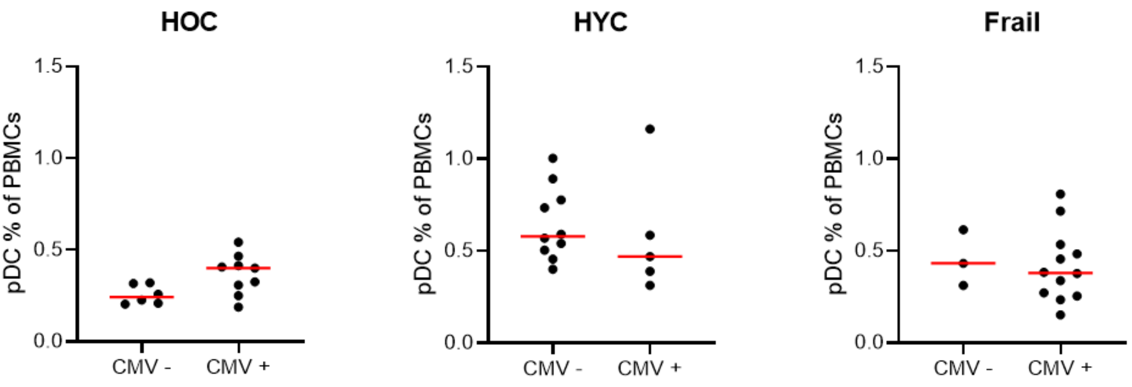

Supplementary figure 3: Proportion of pDCs within total PBMCs in older healthy controls (HOC), young healthy controls (HYC) and Frail participants. The red line represents the median. Mann Whitney U tests showed no statistically significant differences.

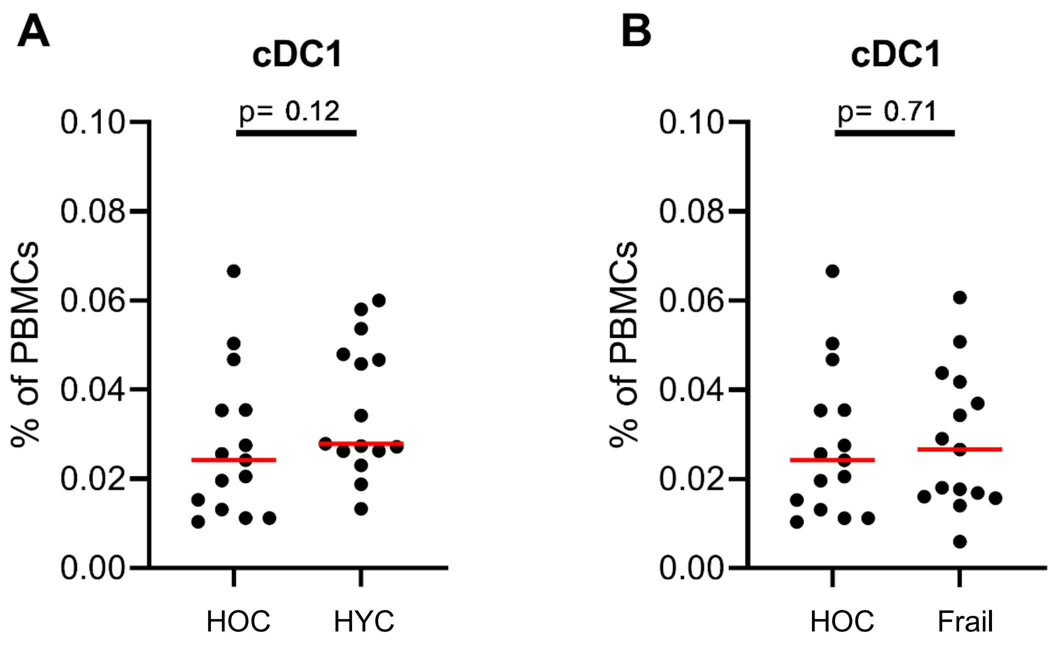

Supplementary figure 4: Proportion of cDC1 cells within total PBMCs. A: Proportion of cDC1 cells in older healthy controls (HOC) and young healthy controls (HYC). B: Proportion of cDC1 cells in older HOC and frail donors. The red line represents the median, and p-values of the Mann Whitney U test are shown in the graphs.

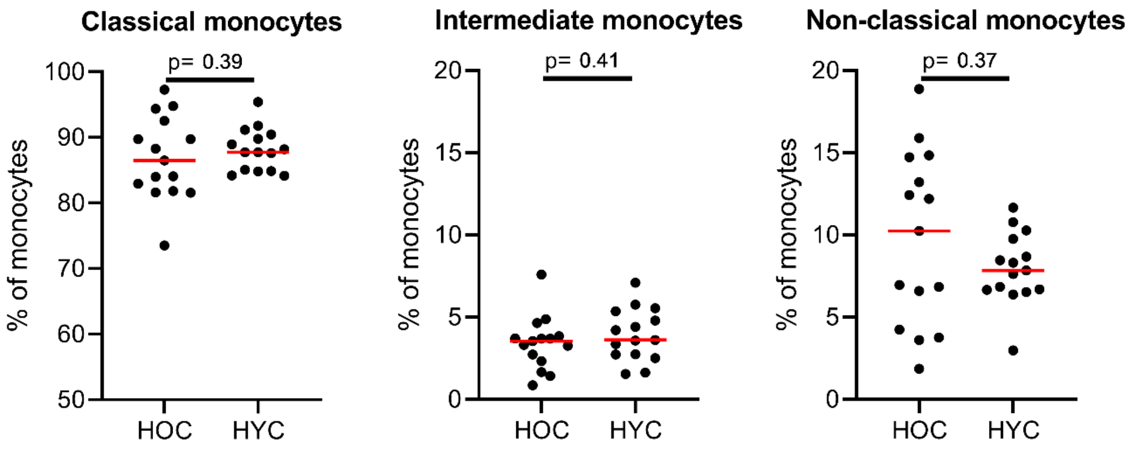

Supplementary figure 5: Proportion of monocyte subsets of total monocytes for the HOC and HYC groups. The red line represents the median, and p-values of the Mann Whitney U test are shown in the graphs. No significant differences between the HOC and HYC groups were found, indicating no evidence for a shift within monocyte subsets with age. HOC: healthy control, HYC: healthy young control.

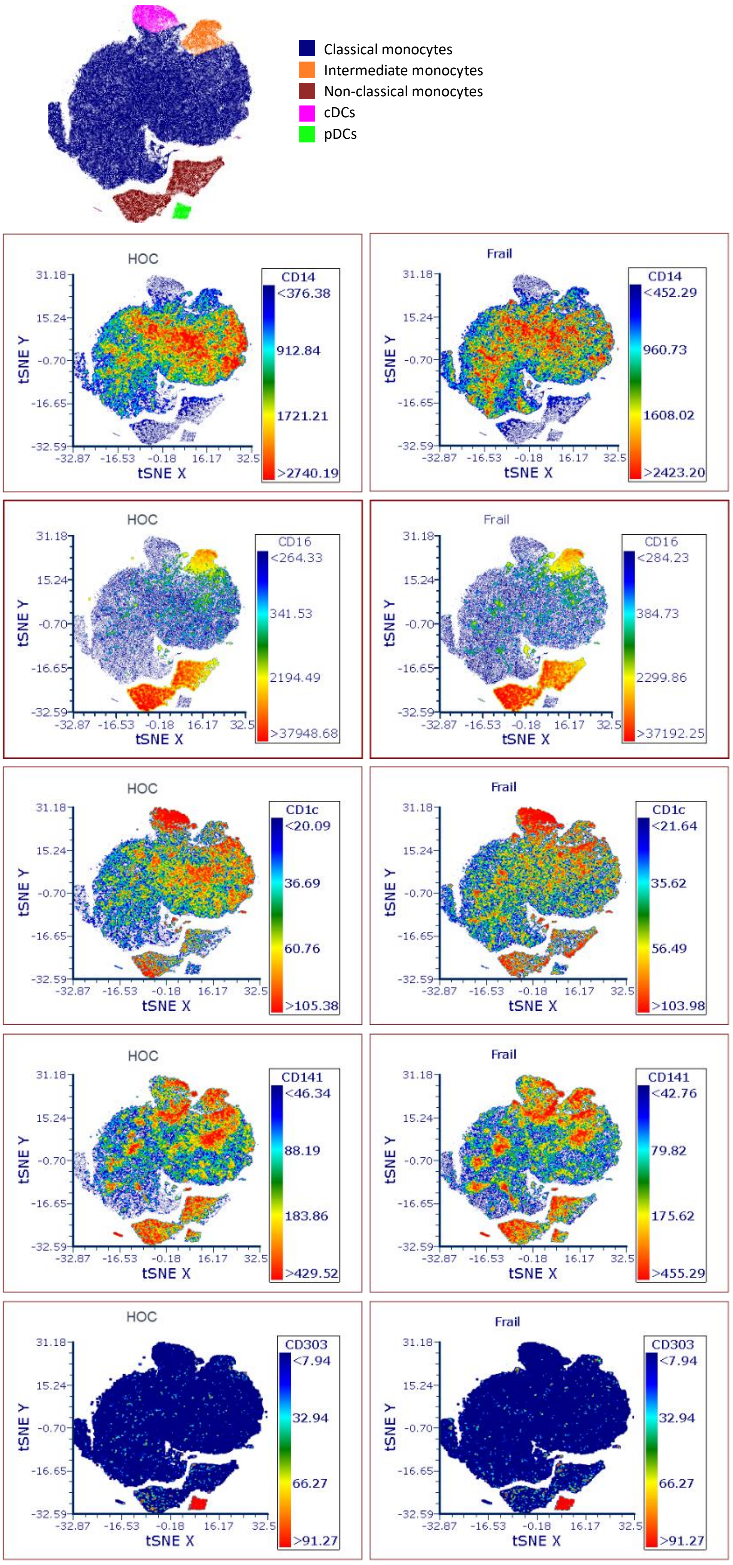

Supplementary figure 6: Expression of lineage markers CD14, CD16, CD1c, CD141 and CD303 in t-SNE plots of healthy older controls (HOC) and frail donors for the identification of clusters with non-classical monocytes (CD14lowCD16+), intermediate monocytes (CD16+CD14+), classical monocytes (CD14+CD16-), conventional dendritic cells (CD141/CD1c+) and plasmacytoid dendritic cells (CD303+). t-SNE: t-distributed Stochastic Neighbor Embedding.

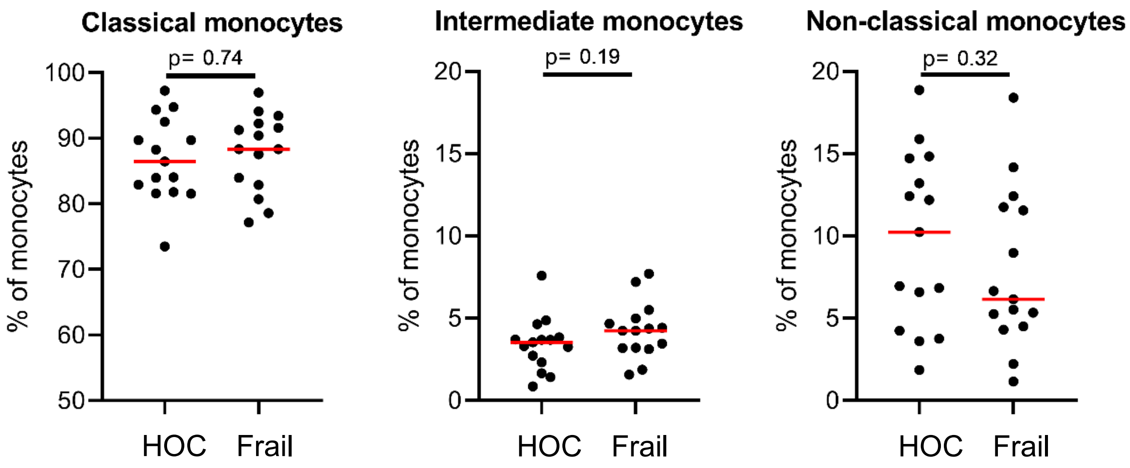

Supplementary figure 7: Proportion of monocyte subsets of total monocytes for the HOC and Frail groups. The red line represents the median, and p-values of the Mann Whitney U test are shown in the graphs. No significant differences between the HOC and Frail groups were found, indicating that in frail older people there is no evidence for a shift within monocyte subsets. HOC: healthy control.
